# Staying at Work with Musculoskeletal Pain in a Physically Demanding Job: A Qualitative Exploration of Workers’ and Managers’ Perspectives

**DOI:** 10.1007/s10926-025-10329-9

**Published:** 2025-09-03

**Authors:** Mette Jensen Stochkendahl, Charlotte Brøgger Bond, Jan Hartvigsen

**Affiliations:** 1 Center for Muscle and Joint Health, Department of Sports Science and Clinical Biomechanics, University of Southern Denmark, Odense, Denmark; 2 Chiropractic Knowledge Hub, Odense, Denmark

**Keywords:** Stay-at-work, Musculoskeletal, Workplace, Workers, Managers, Qualitative

## Abstract

**Purpose:**

Staying at work despite recurrent or persistent musculoskeletal (MSK) pain offers mental and physical health benefits in addition to financial security for workers. This study explores worker and manager experiences and perspectives on stay-at-work practices to nuance our understanding of what drives successful or unsuccessful practices.

**Methods:**

We conducted a vignette-based qualitative exploration of workplace practices of managers and workers from three medium-sized workplaces with physically demanding jobs. Data collection included five focus group interviews and two workshops. Data were analysed using reflexive thematic analysis inductively and deductively.

**Results:**

We identified three themes relating to workplace cultures (Theme 1. Pain is an accepted part of working life), conflicting perceptions of responsibility in managing health impact on stay-at-work practices (Theme 2. Stay-at-work responsibilities and practices), and the importance of trusting communication and the precarious balance between support and pressure to stay at work within the workplace (Theme 3. En route to dismissal).

**Conclusion:**

The identified themes describe helpful and unhelpful beliefs and practices involving individual workers, their co-workers and managers. Shared and unshared cultural norms, understandings of what MSK pain is and means, and perceptions of responsibility drive stay-at-work practices, with trust being an important factor for the precarious balance between workplace support and pressure to stay at work.

**Supplementary Information:**

The online version contains supplementary material available at https://doi.org/10.1007/s10926-025-10329-9.

## Introduction

Many workers in physically demanding jobs experience musculoskeletal pain (MSK), which affects their work life and threatens their ability to remain active in the labour market [1–3]. For example, workers in the Danish construction and cleaning sectors have up to 40% higher prevalence of back pain compared to office workers and academics [4], and those with persistent or recurrent pain and functional limitations face an increased risk of short- and long-term sickness absence as well as premature retirement [5, 6].

Being able to and wanting to stay at work despite recurrent or persistent pain offers mental and physical health benefits in addition to financial security for individuals [5, 7]. It helps prevent the loss of labour force participation and mitigates the rise in welfare-related expenditures, which presents a significant challenge to sustaining welfare programs in many countries [7].

MSK pain-related work disability has a multifactorial origin, and the specific cause mostly remains elusive [8–10]. Workers with MSK pain experience persistent or recurrent pain episodes, with variations in pain intensity and energy levels depending on their work and family life situations [11–13]. Such fluctuating and often unpredictable symptoms can negatively affect staying at work [14], and persistent pain is associated with lower vocational fulfilment and feelings of being ostracised in the workplace [5]. Most workers with MSK pain, however, cope with their situation and maintain employment, and those who remain at work report lower levels of emotional distress, perceived workloads and physical disability, and higher pain acceptance, life control, and pain self-efficacy [14–16].

Workplace social support from co-workers and employers is a key factor in workers’ decisions to stay at work [15, 17, 18]. Therefore, a worker’s immediate supervisor or manager is crucial in initiating stay-at-work procedures when work participation is threatened [19, 20], and is often best positioned to facilitate work adjustments and communication [21, 22]. Such adjustments may be challenging in many physically demanding jobs and production lines, where it can be difficult to find adjustments that meet both the worker's needs and production requirements [23].

In response to changes in population demographics and the risk of future labour shortages, the Organisation for Economic Co-operation and Development emphasises the importance of preventing, rather than reacting to, sickness absence and work disability [24]. In Denmark, the national legislation already stipulates a wide range of obligations and opportunities for accommodating workers with MSK pain within the welfare state model of *Flexicurity* (A tax-funded all-cause compensation system with high levels of social security combined with labour market flexibility). The Flexicurity model gives workplaces the flexibility to hire and dismiss workers easily, but for workers, it can mean low job security and risk of dismissal, especially in cases of sickness absence or inability to perform a task, depending on collective agreements.

To develop sustainable tools at both individual and organisational levels that help workers and managers achieve long-term employment for workers with MSK pain in physically demanding jobs, we must first understand current workplace inclusion practices. This includes examining perspectives from both workers and managers to identify the helpful practices and those that are not. This study explores worker and manager experiences and perspectives on stay-at-work practices to nuance our understanding of what drives successful or unsuccessful practices.

## Methods

### Design

We included managers and workers from three workplaces with physically demanding jobs to qualitatively explore their experiences and perspectives on stay-at-work practices. Between March and December 2018, we conducted five focus group interviews with managers and workers and two workshops with workers on their stay-at-work practices.

The focus groups were conducted to understand participants’ experiences and perspectives and elucidate both shared meanings and diverse viewpoints in a time-efficient manner. At one worksite and after dialogue with the executive manager of the facility services, we took a participatory approach to mutually benefit both researchers (getting data) and the workers (generating collective discussions, reflections, and solutions). The workshop format allowed for more participants and wider perspectives, thus adding to the depth of the findings from the focus group interviews. Comparable weights of credibility, relevance, and richness were assigned to all data sources.

This study was reported using the consolidated criteria for reporting qualitative research (COREQ) study [25] (Online resource 1).

### Participants

We aimed to include a purposive sample of workers as well as managers from workplaces in sectors with physically demanding jobs, facilitated by union and employer association representatives from a stakeholder group involved in the project. They identified and contacted higher-level managers at three workplaces, who agreed to participate. Snowball sampling was then used to recruit workers with MSK pain and managers for the focus groups, as well as a mixed group of workers with and without MSK pain for the workshops. Participants were provided with detailed written and verbal information about the study's aim and content. The date and time for focus group interviews were set based on availability, whereas workshops were conducted as participatory staff events on dates determined by management.

Participants came from three medium-sized workplaces (50–500 workers), all part of larger companies: (a) a public sector facility service team (primarily cleaning), (b) a distribution centre for non-food goods to supermarkets, and (c) an abattoir. Three focus group interviews, with the participation of eight managers and one HR representative, were conducted (one at each of the three workplaces), and two focus group interviews (one group from each of the abattoir and distribution centre) with nine workers with MSK. In the workshops, the entire staff of approximately 65 persons was invited to participate during working hours as part of a workplace event. Only workers absent on the days of the workshops did not attend. The characteristics of the participants in the focus groups are detailed in Table 1. We employed Malterud’s [26] concept of information power to guide the sample size, needing fewer participants by having clear research objectives (exploring stay-at-work practices), a specific sample (first-hand experiences of working with or managing MSK problems), and a clear analytic strategy (reflexive thematic analysis), but more participants to account for the lack of theoretical underpinnings and the quality of the dialogue (partly inexperienced interviewers). The sample size of the entire data set (focus group interviews and workshops) was deemed to have enough information power to provide a rich and relevant data set adequate to answer our research question [26].

**Table 1:** Characteristics of focus group participants

	Workplace	Position	Gender	Age group
Interview 1	Distribution centre			
	M1	Team manager	Male	30s
	M2	Team manager	Male	40s
	M2	HR consultant	Female	40s
Interview 2	Facility services			
	M4	Team manager	Female	40s
	M5	Team manager	Female	40s
	M6	Department manager	Female	50s
Interview 3	Abattoir			
	M7	Team manager	Male	50s
	M8	Team manager	Male	50s
	M9	Team manager	Male	50s
Interview 4	Distribution centre			
	W1	Worker	Male	40s
	W2	Worker	Male	50s
	W3	Worker	Male	50s
	W4	Worker	Male	50s
	W5	Worker	Male	50s
	W6	Worker	Female	50s
Interview 5	Abattoir			
	W7	Worker	Female	50s
	W8	Worker	Male	50s
	W9	Worker	Male	40s
Workshops	Facility services			
	Workshops held on 2 separate occasions	All workers in the facility service team, approximately 65 workers	Approximately 75% female	20s–60s

### Data Collection

Interview data were collected using focus group interviews to encourage discussion and interaction among participants, aiming to uncover detailed descriptions and the sharing of viewpoints on stay-at-work practices [27]. The focus groups followed a semi-structured interview guide developed by the authors and inspired by the concept of Organisational Health Literacy (Online resource 2). The concept is an integrated approach to understanding health behaviour in organisational settings and has been suggested as a suitable framework for addressing individual, interpersonal, and organisational level factors simultaneously in the workplace [28]. Topics included: (1) Experiences of current stay-at-work practices at an individual level; (2) Worker-manager communication and interactions relating to stay-at-work practices; and (3) Interactions with other stakeholders.

**Fig. 1:**
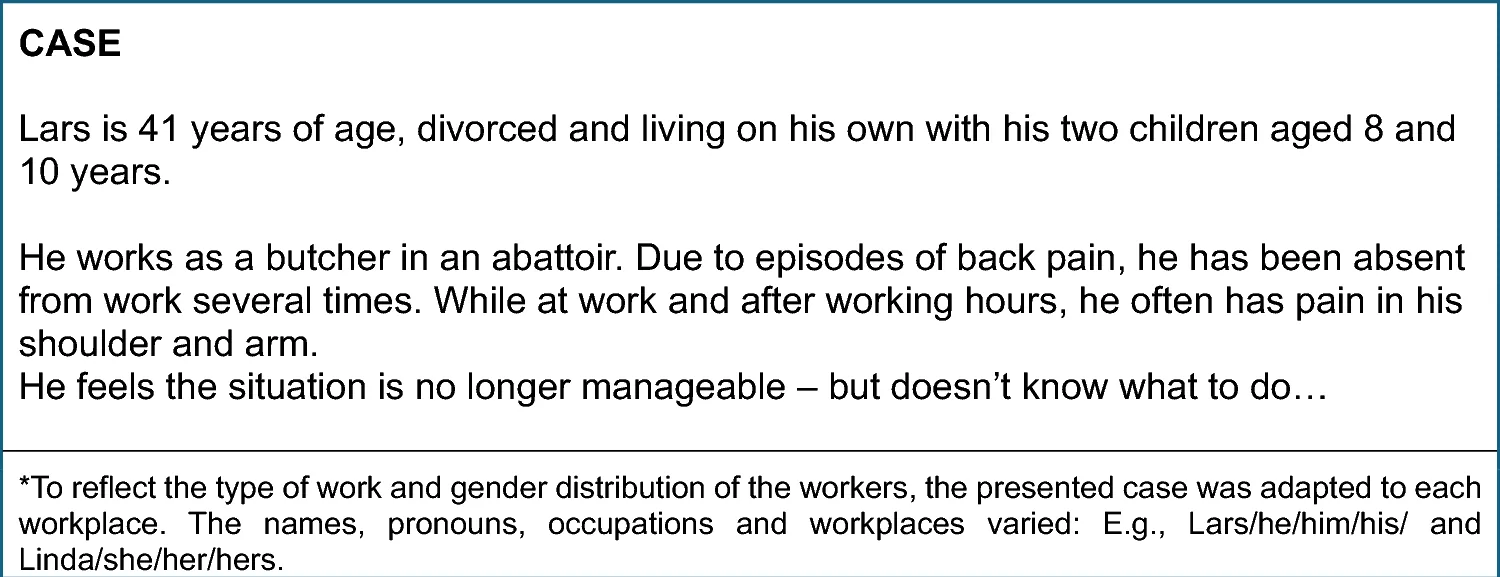
Exemplary case vignette*

In both the focus group interviews and the workshops, participants were presented with a short case vignette illustrating a worker struggling to stay at work due to MSK pain (Fig. 1). The vignette was designed by the authors to reflect common stay-at-work situations and illustrate important predictive factors of sickness absence. The vignette provided a common foundation for the discussions and ensured consistency across all participant groups [29].

Each focus group interview lasted approximately 1.5 h and was conducted in Danish at the workplace, audio-recorded and transcribed verbatim. The manager groups were moderated by the principal author (MJS), a researcher experienced in qualitative research. The worker groups were moderated by pairs of two novice researchers (two master's thesis students without experience, and a PhD student and a research assistant, both with one year of experience). Before the focus group interviews, the novice researchers were trained by the principal author and an experienced colleague in interview techniques and facilitation of group interviews. Open-ended questions were used to facilitate discussion, with examples provided as trigger questions and prompts in the interview guide.

The workshops were facilitated by MJS and assisted by three researchers /research assistants. They lasted three hours. The facilitators aimed to create a supportive atmosphere that encouraged participants to share their experiences and viewpoints, including those that might be conflicting [27]. In the first workshop, workers were asked to identify and prioritise challenges in stay-at-work practices. In the second workshop, the focus shifted to identifying and prioritising effective stay-at-work practices, including good communication practices.

Both workshops began with a plenary introduction to the topic. Afterwards, the workers split into four groups of approximately 15 workers each, who were assigned a facilitator and presented with the vignette. Group participants had the opportunity to work in pairs, in smaller groups, or all together.

In the first workshop, participants brainstormed challenges and used sticky notes and posters to document their ideas. Graphic facilitation was used in the second workshop to aid discussions [30]. Pre-prepared posters with questions were handed to participants, who were asked to reflect on the questions using drawings, cut-outs from newspapers and magazines, and text. The end products were visualisations of the workers' joint knowledge, experiences, and ideas about stay-at-work practices [30]. The shared output helped us understand the meaningful and important factors that drive the success and failure of the stay-at-work practices. In both workshops, successes and failures were discussed and prioritised in the groups. The process and findings were documented using photos of participants' outputs and researchers’ field notes. Following the same topics as the interview guide for the focus group interviews, the research assistants were asked to summarise the discussions and provide their observations and reflections on the group discussions.

Finally, the data generated in the interviews and workshops were discussed with the union and employer representatives in the project’s stakeholder group to elucidate recognition of and reflection on the findings.

### Data Analysis

The lead analyst of the data (MJS) is an experienced researcher knowledgeable of return-to-work practices and work disability prevention, and with previous clinical experience with MSK pain patients. Throughout the analysis and in an iterative process, MJS consulted with CBB, a doctoral student with qualitative expertise within occupational health research, and JH, an experienced MSK pain clinician and researcher, to discuss and refine the emergent themes. The data were analysed through reflexive thematic analysis (TA) used inductively and deductively [31, 32]. Reflexive TA allows for the development of patterns of meaning across data sets that address research questions related to people's experiences, practices, and behaviours, and the factors and social processes that influence and shape particular phenomena, like staying at work. The six phases of Reflexive TA were conducted recursively, with movement back and forth between different phases. Details of the analytic process are presented in Table 2.

**Table 2:** The analytic process

Analytic steps	Phase 1. Familiarisation with the data	Phase 2. Semantic coding of excerpts	Phase 3. Generating initial themes	Phase 4. Developing and reviewing themes	Phase 5. Refining, defining, and naming themes	Phase 6. Writing up
Analytic process	Reading and re-reading transcripts and listening to audio files to become deeply familiar with the data. Making notes on analytic ideas and insights	Labelling of segments of data to indicate patterns of meaning	Clustering codes of shared meaning in an initial analytic framework	Revision and refinement of initial themes	Defining and naming themes; providing structure and analytic flow through the final narrative	Transforming informal writing to formal reporting of results
Conducting the analysis	MJS read the transcripts and notes, inspected photos and posters and noted initial impressions Initial impressions were discussed with the focus group and workshop facilitators	MJS coded data segments inductively using semantic labels close to the participants’ language Codes were compared and discussed with two research assistants involved in the study	MJS clustered codes of shared meaning across interviews and wrote topic summaries MJS made visual maps of candidate themes and relations between themes	MJS wrote initial narratives describing the themes and selected excerpts. CBB and JH discussed and commented on the narrative to refine the initial themes	MJS wrote definitions and boundaries of the themes and their contributions to the overall analysis. CBB and JH commented	MJS wrote the analytic narratives and selected excerpts together with CBB and JH
Software tools used	Electronic and paper files of transcripts, photos and posters	NVivo	NVivo Word Mind maps	Word	Word	Word

In the results section, we present the results of the reflexive process with excerpts from workers and managers and the researchers’ field notes substantiating our interpretations, thus combining the participants’ words with the analytic findings. We have selected excerpts to clearly and richly illustrate the themes rather than validate our findings [33]. Although some participants are more extensively represented due to their clarity of voice, similar perspectives were expressed by other participants across all cases, indicating thematic consistency. The excerpts and field notes were translated from Danish to English by the research team and professional translators to ensure meaning [34]. Participant pseudonyms are used throughout (worker (W), manager (M)).

## Results

The results are presented through three main themes, with two, two and three subthemes, respectively (Table 3).

**Table 3:** Overview of themes and subthemes

Themes and subthemes	Theme characteristics
Theme 1. Pain is an accepted part of working life	
1.1 Contrasting perceptions of the cause of pain 1.2 Balancing work performance and health	Theme 1 describes the workplace actors’ understanding of what MSK pain is and means, working in a physically demanding job, and the underlying workplace cultures and norms that impact stay-at-work practices and actions
Theme 2. Stay-at-work practices and responsibilities	
2.1 Assuming responsibility for staying at work and using assistive devices 2.2 Knowledge and trust are important for gaining access to workplace resources	Theme 2 describes managers’ and workers’ perceived roles and responsibilities in stay-at-work practices and how knowledge and trust are important factors that facilitate help and support
Theme 3. En route to dismissal	
3.1 Healthcare providers’ role in stay-at-work processes 3.2 To disclose or not disclose 3.3 The potential threat of engaging in care dialogue towards staying at work	Theme 3 describes the involvement of healthcare providers in stay-at-work processes, the workers’ risk of disclosing their condition and the dialogues that unfold to either facilitate staying at work or lead to the dismissal of the worker

*MSK* musculoskeletal pain

### Theme 1: Pain is an Accepted Part of Working Life

#### Contrasting Perceptions of the Cause of Pain

The workers identified well with the description in the vignette, “*I’m not actually called Lars [name of the fictive worker in the vignette], but you’re pretty close.”* (W9). The excerpt highlights how MSK pain was accepted as a natural consequence associated with having a physically demanding job.

It [MSK pain] probably can’t be avoided when the work is so physically demanding, but we all have some [aches and pains] here and there from time to time, I think. Some more than others. (W6)

Workers went on to describe how the use of prescribed and non-prescribed pain medication was widespread and accepted as a necessary means to stay at work; “*So I gobble down some painkillers during the workday. I can’t hide that from anyone.”* (W1). In the abattoir, the use of pain medication was openly discussed and accepted among co-workers.

Interviewer: “You talked about how you take a lot of pain medication. So…you just talk about it? Is it something everybody talks about?”. W8: “Yes”. W9: “Yes, I think so”. Interviewer: “So it's an open conversation, there’s nothing you hold back? Your co-workers know about it? Management knows?”. W9: “They know”. W8: “At least [name of manager] knows”.

Workers recognised psychosocial aspects of pain (such as family life situations or mental burdens) as relevant factors for staying at work, but mainly attributed MSK pain to the physically demanding nature and organisation of their work tasks, with limited flexibility allowed for work adjustments and breaks. Workers expressed that there were no easy workstations or tasks, and only limited options to customise existing workstations. They all mentioned working at a high pace under time pressure and constant monitoring to be on target (piecework). Despite mandatory rotation between workstations (abattoir and distribution centre), workers shared the perception that piecework negatively impacted MSK pain and health in general.

Like the workers, managers identified the vignette as representative of their workers, *“We have many with back pain.”* (M6) and acknowledged the demanding nature of the tasks and high prevalence of pain and use of pain medication.

It is hard work on the production line, de-boning, you know, it’s hard on the muscles. It takes a good body, a very good body. (M8)

It's a bit scary, you know? But I actually think you’re right, because there are probably a lot of people who are in pain and take some pills, … and more than we think. (M7)

Contrary to the workers, managers emphasised psychosocial factors outside the workplace as more important underlying or contributing causes of MSK pain.

I often feel it may be a combination… The psychological part is actually caused by something going on at home, but then it becomes work-related because you have to go to work every day…. We know those [workers] who have challenges with mental health really well; they’ve all been very frank about it, and practically all [the problems] have started at home. (M6)

#### Balancing Work Performance and Health

Workers described showing up for work, not saying anything, and working through the pain either to “*work it off*” (sticky note from the workshop) or to avoid taking time off and feeling pressure to perform.

You can choose between taking care of yourself and getting a comment from your supervisor that you haven’t reached your target, or you can choose to push on, reach your target and then you just wear yourself down. (W3)

Many workers expressed pride in their work and a great sense of responsibility towards the workplace and accepted the demanding nature of their tasks, “W*e’ve chosen a physically demanding job, you know, so it can’t come as a surprise to anyone.”* (W3).

For many workers, the work culture had become an integrated part of their lives, and they appreciated the certainty of knowing the workflow and jargon as major reasons for staying. As put forward by one distribution centre worker, *“I can’t imagine anything else, or being anywhere else.”* (W5)*.*

### Theme 2: Stay-at-Work Responsibilities and Practices

#### Assuming Responsibility for Staying at Work and Using Assistive Devices

When experiencing pain, the workers mentioned various options and discussed how they would handle the problem early on, by first and foremost looking out for themselves, both at home and at work. The first included sleeping well, exercising, and taking responsibility for staying mentally and physically healthy. The latter included paying attention to work posture, prioritising or organising their tasks differently, switching tasks with co-workers, or seeking advice from an experienced co-worker or online. Many workers discussed the importance of using assistive devices, but also mentioned that they were not always accessible, and defects or time pressure did not allow their use:

Interviewer: “We heard about suction cups you could use for lifting, but they’ve never really been used…?”. W2: “No, it takes too long to use [them]”. W5: “Then we’d never have been able to reach those numbers [the target] “

In contrast, according to this manager, the (non-) use of an assistive device was mainly up to the worker.

When we offer an assistive device to a worker, I think the challenge is that it’s a hassle for some workers to use it… at a high pace…in repetitive work tasks, getting a device that can hamper them a bit in their work, and then getting them to use it, you know? It’s easier for them to throw it aside, do things manually again. (M7)

While managers placed the responsibility of using the devices on the workers, in these fast-paced workplaces with high performance demands combined with a cultural norm of working through the pain, the workers’ motivation for using devices that slowed them down was low, leaving a vacuum were neither workers nor managers assumed the responsibility of their use.

#### Knowledge and Trust are Important for Gaining Access to Workplace Resources

Many workers said they would prefer flexible or reduced work hours or loads when experiencing MSK pain. However, it was generally perceived that few of these options were available. Most pronounced in the abattoir, the workers expressed that modified work was almost impossible due to the static nature of the linework or lack of accommodation or the manager’s and the company's willingness to incur the costs.

But they [the managers] can’t [offer accommodation] … Well, they can, but it costs a lot of money…. And if I’m the only one brave enough to speak up, about something being wrong, they do nothing. (W8)

Many workers acknowledged the manager's right to distribute work tasks and empathised with the manager when having to make difficult calls on offering lighter duties to some workers at the expense of others. Other workers felt that particular workers were more likely to be offered accommodation and light duties.

We have a few tasks that aren’t so hard, but a few people have already grabbed them, and they [the managers] won’t do anything about it. It’s usually people who’ve been here for many years. (W3)

Managers also felt they had limited options for providing suitable accommodation or modified tasks, and that the solutions could only be of limited duration. Because many employees had MSK pain, they felt hampered in offering solutions and were sometimes met with scepticism and criticism from co-workers.

*The rest of the team says, ‘*Really, now he gets light duties again? He bloody well got that a fortnight ago too; how long can he carry on with that*?’* (M8)

Managers were willing to accommodate all workers, but their solutions were impacted by their feelings towards specific workers. Frequently, there was mistrust, and managers were sometimes led to believe that workers were dishonest and exploiting their right to accommodations or time off work.

So, you know, they [co-workers] notice that it [the lighter duties] gets taken advantage of, you know? And then I’ve said, I won’t bother wasting my time on them over there [who take advantage of the system]. Of course, the policies apply to them too, but I would rather use my time on the people here [the co-workers] and treat them right, talk to them and offer what I can. (M8)

The workers offered accommodation were typically workers with whom the managers worked more closely and therefore, knew well and trusted more.

Interviewer: “When is an employee a good team player?” M5: “When they are willing to cooperate and give things a chance.” M4: “and don’t have that negative attitude that screams 'stay away from me'”, M5: “Yeah, that negative attitude of 'you’re hounding me’ or whatever”.

As described above, the managers conditioned their help on dialogue, trust and collaboration. However, some workers placed the responsibility to find solutions entirely on the managers. This became apparent during one of the workshop sessions with the cleaners when presented with the vignette and asked, “*What do you suggest Linda do in this situation*”?

One group of four people struggles to get started. They don’t understand the question. I ask if they recognise Linda’s situation, and they do. When I rephrase the first question and ask what they would do in a similar situation, they are silent. When further prompted, they tell me that they don’t see that they can do anything at all. It’s up to the manager to do something. (Researcher’s workshop notes)

### Theme 3: En Route to Dismissal

#### Healthcare Providers’ Role in Stay-at-Work Processes

Not all workers turned to outside help: “*We don’t go to the doctor, we use co-workers”* (workshop sticky note)*,* but most workers and managers resorted to involving healthcare providers in stay-at-work processes. Workers in two of the three workplaces had additional health insurance, which gave them access to massage, physiotherapy, and chiropractic services free of charge. These services were highly valued and perceived as important for staying at work, and those without access inquired about them. When approaching their manager with MSK pain, many workers had experienced being met with the question “Have you seen a healthcare provider?”, and the managers often suggested the workers use their health insurance as the first choice of action. They expected the worker to get a diagnosis or referrals to advanced diagnostics and treatment. In case of sickness absence, managers expected a clear return to work (RTW) plan, and if a Declaration of work capacity was required, they expected the general practitioner to get involved and help timeframe RTW.

“That they reach out to [the health insurance], and get it sorted out quickly. What is it, what’s the time horizon?”. (M1)

However, the managers had mixed experience with the involvement of general practitioners and outputs. They had experienced that a general practitioner could delay RTW by suggesting a longer time off work than the manager thought was necessary or was willing to accept, and by not providing a clear timeframe for RTW.

When I get one of those [Declaration of work capacity] that says ‘duration unknown’ when I’m reading that, I think the doctor has just signed the worker’s dismissal papers. (M8)

#### To Disclose or Not Disclose

Many workers were not willing to disclose their MSK pain until they were forced to call in sick. In the abattoir, managers expected the worker to reach out to the manager in case of problems before it resulted in sickness absence.

To some extent, we don’t accept that you [the worker] call in saying you can’t come to work because you’re in pain. Because we have kind of put it on the workers, too, that it is their responsibility to come to us and tell us how their body is doing. (M7)

The workers stated that it was important to talk about MSK pain openly and to be “believed” by managers. Many workers reported worries about what would happen if they talked to the manager. In the workshops, the cleaners wrote sticky notes with phrases like *“afraid to lose your job”, “downward spiral”, “shame”, “bad economy” and “don’t know the consequences”* as potential costs of acting on their pain.

#### The Potential Threat of Engaging in Care Dialogue Towards Staying at Work

In cases of prolonged or repeated episodes of sickness absence, all three workplaces had RTW policies that mandated individual worker-manager meetings, “care dialogues”, at specified times to find suitable solutions. Workers characterised a good care dialogue by the presence of an empathetic manager, trust, confidentiality, and openness about the problem. While some workers had positive experiences with this type of dialogue, others were frustrated when the manager did not follow through with the agreed-upon accommodations. Some workers experienced the care dialogue as a formality that focussed on the consequences of low performance or sick leave rather than on worker well-being. They described not feeling heard, acknowledged, or recognised, which resulted in an increased scepticism towards the manager. In a few cases, the dialogue was even perceived as coercive.

They talk about their own experience. Carpal tunnel syndrome, … a broken arm and demands about returning to work once the cast is off. Everyone in the group expressed frustration about not being believed by the manager. There is mistrust and a lack of understanding. They feel pressured and afraid of losing their jobs. (Researcher’s workshop notes)

Managers described an obligation to follow the company's RTW policies and handle the situation professionally, which included decent, fair and equal treatment of the workers. Managers recognised the workers’ vulnerability in care dialogues and believed that good solutions were best achieved by knowing the workers well and creating an atmosphere of trust. They explained that workplace policies helped create a confidential space for facilitating dialogues about MSK pain, but noted that this perception was not always shared by the workers.

You’ve [looks at DCM3] recently had a case with a worker who certainly didn’t think we meant it. And who was very quick off the mark and wrote a text message to you, I think, with "you can just fire me, just fire me" or something like that. (M1)

They also recognised that the conversation was a balance between helping and putting pressure on the worker. The managers acknowledged that sometimes these conversations served as a warning to the workers, indicating that continued sick leave or requests for work accommodations could have consequences.

Like you say [directed at M2], ‘it’s just a care dialogue’. But it isn’t just that, because it’s important that our workers understand that there might be consequences, you know. It’s a balance between being caring but also consistent. (M1)

## Discussion

Presented in three themes, we describe the complex interplay between MSK pain, work culture and the actions and practices employed by workers and managers to navigate the challenges of staying at work while managing pain. The themes reveal a shared acceptance of pain as an inherent aspect of working life, the complex and sometimes conflicting perceptions of responsibility in managing health and productivity, and the precarious balance between support and pressure within the workplace.

### Comparison with Previous Findings

MSK pain was largely accepted as a normal part of working life, which aligns with previous research indicating that workers in physically demanding jobs internalise pain as a necessary sacrifice for job security and financial stability [5], and framing it as a trade-off between staying employed and facing unemployment [35]. Christiansen et al. [35] and Ajstrup et al. [36] found that management priorities and group norms around pain acceptance influence both MSK pain and sickness absence; accepting pain as a part of working life may drive some individuals to stay at work while pushing those who do not accept this culture out of the workplace. Christiansen et al. [35] suggest that shared meanings within workgroups can make pain seem manageable or even acceptable. Our findings further show that many workers indirectly accepted the status quo at their workplaces because of valued work outcomes other than income, such as social status, purpose in life, and camaraderie. In this perspective, health is considered a resource, and employment is the state individuals want to preserve [37], sometimes despite the burden of suboptimal health [5, 37, 38].

Workers' and managers' perceptions of the cause of pain, and, consequently, the solutions they pointed towards, and the responsibility to enact the solutions, differed. Similar to previous findings [39], workers in our study described proactive strategies and the importance of staying healthy, yet predominantly blamed workplace exposures as the cause of pain. They expected their workplace to be accommodating, but also often sought medical care or ignored pain in fear of reprisal. Managers, in contrast, pointed towards non-work psychosocial factors and worker responsibilities to stay healthy. Hence, for workers, ‘responsibility’ means the autonomy to decide on engaging in healthy behaviours, while managers equate ‘responsibility’ with workers’ duty to stay healthy [40, 41].

The prevalence of MSK problems is generally underestimated [42–44], resulting in a lack of systematic management [43, 45]. The normalisation of pain and an emphasis on workers' obligation to stay fit and healthy suggest a shift in the burden away from the workplace’s responsibility. The managers expressed responsibility for workers' health by providing suitable work accommodations and showing an interest in their wellbeing; however, as seen elsewhere in the literature [46, 47], they perceived their responsibility as limited by budgets, production constraints and consideration of the whole work team. Accommodation was offered temporarily, with the expectation of a fast return to full duties, and more willingly to those perceived as “good workers”.

Workers face dilemmas related to disclosing their conditions to the manager, which is a barrier for some to engage in constructive and solution-seeking dialogues. Workplace practices are highly dependent on the worker’s disclosure of their health problem and needs, co-workers' perception of the worker, and the length of the health problem [15]. In an effort to uphold a self-image as a good worker, some choose not to disclose their condition and deny symptoms, work their way through the pain, and take leave to cope with the pain [12, 13, 16, 48]. Managers in our study perceived this strategy negatively, and workers risk overstepping an invisible line where they lose the support of their manager. The care dialogues mandated by workplace policies represent an attempt to bridge the gap between worker needs and managerial expectations. However, the outcomes of these dialogues were contingent upon the establishment of trust and open communication [13, 45, 49, 50].

### Study Weaknesses and Strengths

We focussed on three workplaces in Denmark, which may not adequately represent the diverse experiences and practices in different organisational, legislative or cultural contexts, reducing the transferability of our findings. Data were collected in 2018. Although the Danish legislation has remained unchanged since then, and it is possible that labour shortage has led employers and organisations to change stay-at-work practices. The use of gatekeepers can lead to the exclusion of workers with differing views or experiences, and we cannot exclude that the distribution of the participants’ gender, age, and being predominantly native Danish has led us to miss important aspects concerning differences across genders, age groups, and immigrant status reported in other studies [51, 52]. Although carefully facilitated and with prompts directed to quieter participants, the use of focus groups and workshops may have led to groupthink, where dominant voices overshadow quieter participants. Finally, the interviewers’ and moderators’ varying levels of experience potentially affect the richness of the data.

These drawbacks are countered by the involvement of numerous researchers in the data collection and analytic processes and the triangulation of data, ensuring rich, nuanced perspectives from both workers and managers with the workshops, and giving priority to workers’ voices. The collaboration with union and employer association representatives enabled access to a diverse range of participants. Using a case vignette to standardise discussions across focus groups and workshops provided a relatable scenario that helped participants engage meaningfully with the topic, ensuring that discussions remained focussed and relevant. The participatory nature of the workshops fostered a collaborative environment that further enhanced the richness of the data collected.

### Implications for Practice

The current stay-at-work practice builds largely on the same principles as RTW practices. With demographic changes, work participation among workers with MSK pain is increasingly important and must be prioritised in workplace health policies and practices. Organisations should foster a culture that prioritises worker well-being and openly addresses the challenges associated with MSK pain. This includes implementing comprehensive stay-at-work programs that provide a supportive work environment where workers feel safe disclosing health issues without fear of repercussion, and proactive development of feasible and affordable strategies targeting worker accommodations that match both production and workers’ needs.

## Conclusion

Among workers with MSK pain in physically demanding jobs, we identified themes that describe both successful and unhelpful practices involving individual workers, their co-workers and managers. Shared and unshared cultural norms, understandings of MSK pain, and perceptions of responsibility drive stay-at-work practices, with trust being an important factor for the precarious balance between workplace support and pressure to stay at work.

## Supplementary Information

Below is the link to the electronic supplementary material.

Supplementary file1 (PDF 482 KB)

Supplementary file2 (DOCX 40 KB)

## Data Availability

Due to the European Data Protection Regulations, the dataset generated during the current study is not available for additional analysis.
