# Supplementary material for: Staying at Work with Musculoskeletal Pain in a Physically Demanding Job: A Qualitative Exploration of Workers’ and Managers’ Perspectives
Source: J Occup Rehabil. 2025 Sep 3;36(4):1051–62. doi: 10.1007/s10926-025-10329-9 (PMC13759558; doi:10.1007/s10926-025-10329-9)
Supplement: Supplementary file 2 [file 10926_2025_10329_MOESM2_ESM.docx]

# Online resource 2 – interview guides.

Exemplary interview guide for a focus group interview with **workers**.

| **Construct** | **Questions** | **Prompts** |
| --- | --- | --- |
| **Opening questions** | Thinking about your own life, how do you recognise the situation described in the vignette? | - Do you recognize the situation and/or worker characteristics? - How is the vignette similar/dissimilar to your experience/situation? |
| **Current practice** | If you were Lars, what would you do?  What do you think will be the outcomes of the actions?  What do you think will happen to Lars?  Why do you think Lars would act like this? | - What did you do in your situation(s)? How did you know what to do? - If you have had a co-worker in a similar situation, what did they do? - Can you give examples of what you do in situations similar to Lars’s? - In your experience, what happens next/ how did you react to this? - What would you identify as important for a successful/unsuccessful interaction manager? Can you give examples of successful/unsuccessful turn of events? |
| **Communication and interaction with the manager** | In your experience, how did you interact with your manager in this type of situation? | - What did your manager say/do? Can you give examples? - How did you react? How did this make you feel? - Did you know what to do? - What would you identify as important for a successful/unsuccessful communication/interaction manager? Can you give examples of successful/unsuccessful turn of events? |
| **Interaction with other stakeholders** | Who would you identify as essential people that you have interacted with in Linda’s/similar situation? | - Who else might be involved in a similar situation? - Who else have you interacted with? - What makes them important/essential? - How do you interact with them? - What was the outcome of the interaction? |
| **Closing** | Is there anything you would like to add to the discussion?  Is there anything else that you think would be helpful for us to know |  |

Exemplary interview guide for a focus group interview with **managers**.

| **Construct** | **Questions** | **Prompts** |
| --- | --- | --- |
| **Opening questions** | Thinking about your employees, how do you recognise the situation described in the vignette? | - Do you recognize the situation and/or worker characteristics? - Can you give a recent example of similar/dissimilar situations? - How is the vignette similar/dissimilar to your experience? |
| **Current practice** | If Linda were your employee, how would you handle the situation?  What do you think will happen to Linda/an employee in a similar situation? | - What are your usual procedures in this type of situation? - Can you give examples of what you do in situations similar to Linda’s? - In your experience, what happens next/ how is this received by the worker? - What would you identify as important for a successful/unsuccessful interaction with Linda/similar worker? Can you give examples of successful/unsuccessful turn of events? |
| **Worker characteristics** | In your experience, who would you identify as being challenged with staying at work? | - What would be a typical worker that you would most likely identify as having problems with staying at work? - What type of complaints/problems/specific characteristics does the worker have? |
| **Interaction with other stakeholders** | Who would you identify as essential people that you have interacted with in Linda’s/similar situation? | - Who else might be involved in a similar situation? - What makes them important/essential? - How do you interact with them? - What is the outcome of the interaction? |
| **Closing** | Is there anything you would like to add to the discussion?  Is there anything else that you think would be helpful for us to know |  |
